# The bottom-up information transfer process and top-down attention control underlying tonal working memory

**DOI:** 10.3389/fnins.2022.935120

**Published:** 2022-08-01

**Authors:** Qiang Li, Dinghong Gong, Yuan Zhang, Hongyi Zhang, Guangyuan Liu

**Affiliations:** ^1^College of Education Science, Guizhou Education University, Guiyang, China; ^2^Office of Academic Affairs, Guizhou Education University, Guiyang, China; ^3^College of Electronic and Information Engineering, Southwest University, Chongqing, China

**Keywords:** tonal working memory load, sf-MVPA, cortical activation pattern, functional connectivity, attention

## Abstract

Tonal working memory has been less investigated by neuropsychological and neuroimaging studies and even less in terms of tonal working memory load. In this study, we analyzed the dynamic cortical processing process of tonal working memory with an original surface-space-based multivariate pattern analysis (sf-MVPA) method and found that this process constituted a bottom-up information transfer process. Then, the local cortical activity pattern, local cortical response strength, and cortical functional connectivity under different tonal working memory loads were investigated. No brain area’s local activity pattern or response strength was significantly different under different memory loads. Meanwhile, the interactions between the auditory cortex (AC) and an attention control network were linearly correlated with the memory load. This finding shows that the neural mechanism underlying the tonal working memory load does not arise from changes in local activity patterns or changes in the local response strength, but from top-down attention control. Our results indicate that the implementation of tonal working memory is based on the cooperation of the bottom-up information transfer process and top-down attention control.

## Highlights

- A surface-space-based multivariate pattern analysis method is proposed.
- Using this method, the dynamic cortical processing process of tonal working memory is revealed, which constitutes a bottom-up information transfer process.
- The neural mechanism underlying tonal working memory load arises from the top-down attention control of the working memory system.
- The realization of tonal working memory is based on the cooperation of the bottom-up information transfer process and top-down attention control.

## Introduction

Working memory plays a central role in human cognition (Cai et al., 2021) and has been an important issue since it was first proposed (D’Esposito and Postle, 2015). Tonal working memory refers to actively and temporarily maintaining tones in the mind (Gorow, 2011; Galeano Weber et al., 2017), while working memory load refers to the number of items held in working memory (Pinotsis et al., 2019). As an important part of the auditory working memory (Pechmann and Mohr, 1992; Berz, 1995), tonal working memory is essential for musical cognition (Schulze and Koelsch, 2012). However, compared with visual/spatial and verbal studies, tonal working memory has been less investigated by neuropsychological and neuroimaging studies (Daligault, 2017) and even less in terms of tonal working memory load (Grimault, 2014).

In an early functional magnetic resonance imaging (fMRI) study, Gaab et al. (2003) found that the neural basis of tonal working memory involved the superior temporal gyrus (STG), supramarginal gyrus (SMG), inferior frontal gyrus (IFG), precentral gyrus (PCG), superior parietal regions, and dorsolateral cerebellar regions. Using a decoding method, Uluç et al. (2018) found distinguishable neural coding in STG, PCG, and supplementary motor area (SMA) in a content-specific tonal working memory study. In an fMRI study (Schulze et al., 2011) comparing verbal and tonal working memory, Broca’s area, premotor cortex (PMC), pre-SMA/SMA, left insular cortex, and inferior parietal lobe (IPL) were found to be involved in both verbal and tonal working memory. In another verbal and tonal working memory study (Koelsch, 2009) during rehearsal and articulatory suppression, the planum temporale (PT), PMC, IPL, Broca’s area, the anterior insula, and some subcortical structures were found to be activated during a rehearsal of verbal and tonal information. When studying the local neural pattern of working memory of tones, Kumar (2016) found that patterns of activity in the auditory cortex (AC) and left IFG distinguished the tone that was maintained in working memory.

Although a consensus is gradually emerging that a frontoparietal network (Gaab et al., 2003; Schulze et al., 2011; Schulze and Koelsch, 2012; Albouy, 2019; Czoschke et al., 2021) consisting of the inferior parietal lobule (IPL), cerebellum, PT, Broca’s area, and PMC constitutes the neural basis underlying tonal working memory, the dynamic neural processing process of tonal working memory remains largely unknown. In addition, although there has been some electroencephalography (EEG)/magnetoencephalography (MEG) research (Guimond, 2011; Nolden, 2013; Grimault, 2014) concerning the tonal working memory load, due to the limited spatial resolution of EEG/MEG (Ahlfors and Simpson, 2004), the neural correlates of tonal working memory load remain unclear.

Some fMRI studies (Gaab et al., 2003; Albouy, 2019) have attempted to investigate the dynamic processing process of tonal working memory by shifting the scanning time during the memory retention period; however, due to the poor temporal resolution of traditional data analysis methods, the dynamic processing process has not been clearly revealed. In addition to fMRI, local field potential (LFP) is also a potential method to explore the dynamic processing process underlying working memory (Pinotsis et al., 2019; Kumar, 2021). However, despite the high spatial and temporal resolution of LFP, it is difficult to cover the whole cortex with electrodes and is hard to use this method in healthy people, which limits the scope of the method. A more efficient and non-invasive method is needed to reveal the dynamic neural processing process of tonal working memory.

Volume-space-based MVPA mainly depends on a searchlight method (Kriegeskorte et al., 2006) to select voxels that represent the local pattern space. In this method, for a certain central voxel, a spherical-volumetric region around that voxel is defined and the voxels inside that sphere constitute the local pattern space. This spherical-volumetric searchlight method has several flaws (Chen, 2011; Oosterhof et al., 2011). First, the inclusion of non-gray matter, such as white matter, cerebral spinal fluid, and other tissues in a volumetric searchlight, is likely to increase the noise-to-signal ratio. Second, considering the folded nature of the cortex, it is very possible to include remote (in the geodesic distance) non-associated brain areas in a searchlight. Searchlights close to the longitudinal fissure might even include voxels from the other hemisphere.

To improve the defects of volume-space-based MVPA, we proposed a new original surface-space-based MVPA method. This method directly extracts searchlight from the surface space, thus avoiding the disassociation problem and the noise of non-gray matter. Using this sf-MVPA method, we successfully revealed the dynamic cortical processing process of tonal working memory and found that this process constituted a bottom-up information transfer process. Furthermore, we analyzed the local cortical activity pattern, local cortical response strength, and cortical functional connectivity under different tonal working memory loads. We found that the interactions between the AC and an attention control network, consisting of the prefrontal cortex (PFC), posterior cingulate cortex (PCC), and precuneus, were linearly correlated with memory load, demonstrating top-down attention control during tonal working memory.

## Materials and methods

### Participants

A total of 23 young and healthy volunteers with normal hearing (18–23 years, mean age 20.8 years, 11 females, right-handed) participated in this experiment. All volunteers were students of Southwest University (China). None of the volunteers had any extra-musical training beyond general school education. Volunteers were paid for their participation and signed informed consent forms. The experimental paradigm was approved by the Ethics Committee of Southwest University (project number: H21053).

### Experimental paradigm

There were five MRI runs in total. The first run was used to acquire T1 structure data, and during this run, no task was performed. The second run was used to adjust the system volume by the subjects, and during this run, the T2* functional data were scanned for 1 min to simulate the real experimental circumstance. During this run, a song was played *via* headphones, and the subjects were asked to adjust the system volume as much as possible on the premise that they felt comfortable. Foam inner ear plugs were worn by subjects to reduce the noise from the scanner. A formal experiment was performed in the last three MRI runs. Subjects were instructed to keep their eyes open and look at a fixed cross in the center of the screen during the formal experiment. A trial started with a second of silence, and then, a fixed cross was presented in the center of the screen until the answer period. Then, a sequence of piano tones (1–4 in number) was presented to subjects *via* headphones. After a delay period of 20 s, another sequence of piano tones (the same length) was played. Subsequently, the text “were they the same” in Chinese and two buttons, one with the word “same” and another with the word “different,” were displayed on the screen. Subjects had 4 s to answer whether these two sequences were the same with a panel. The panel had two buttons and was held by subjects with the left (12 subjects) or the right (11 subjects) hand. The panel was also used to adjust the system volume in the second run. Subjects were asked to press the buttons with their index finger and the answer was shown on the screen in real time. Under the control condition, during which nothing was played, subjects were asked to press the same button during the answer period. Before entering the scanning room, subjects were trained on a computer to familiarize themselves with the experimental procedures.

**FIGURE 1:**
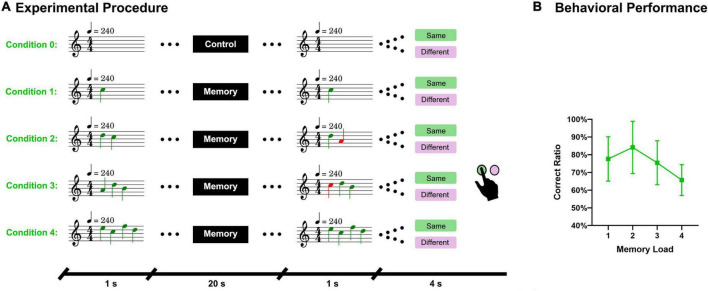
Experimental procedure and behavioral results. **(A)** A sequence of piano tones (1–4 tones) was presented to subjects and after 20 s, another sequence of tones (same length) was presented. Subjects must answer whether these two sequences are the same. There were five conditions in total. Condition 0 referred to the control task, during which no sequence was presented and subjects had to answer “same” in the answer period. **(B)** Accuracy of the answers under different memory loads.

There were 100 trials in the formal experiment, including 20 control trials. These 100 trials were randomly divided into 3 parts, with 33 trials in the first and second parts and 34 trials in the last part. Each part was presented in an MRI run. Nine tones (C1, D1, E1, F1, G1, A1, B1, C2, and D2) were selected as stimuli according to the key of C major. Sequences of tones consisted of free combinations of these 9 pitches, allowing repetition. Piano tones were chosen because a piano is a common musical instrument and is often used in studies of auditory working memory (Jerde et al., 2011; Herholz et al., 2012; Bigelow et al., 2014). To eliminate the interference of other musical elements, such as rhythm and tempo, all tones were in 4/4 m and 240 bpm. There were 20 sequences under each condition and whether the paired sequences were the same was equiprobable. For the paired sequences that were not the same, there was a tone (at a random location) of the probe sequence that varied in two natural tones (up or down with equal probability). We used MATLAB R2020b^1^ to generate the random code of these sequences and used LilyPond^2^ to generate MIDI files. We then used MATLAB R2020b to convert these MIDI files into.mp3 files (sample rate of 44.1 kHz). Later, Adobe Audition CS6^3^ was used to standardize the volume of the sequences and the song used in the second run according to the peak amplitude. We wrote two VC++ programs using Visual Studio 2010^4^ to present the stimuli and visual content. The first program was used to present a song *via* headphones and receive button messages to adjust the system volume in the second MRI run. The second VC++ program randomly presented the paired sequences to subjects and recorded their button responses. The triggering signal sent by the MRI system ensured that the two VC++ programs were synchronized with the scanning. The experimental paradigm is illustrated in Figure 1A.

### Imaging data acquisition and preprocessing

The MRI data were acquired with a 3T Siemens Prisma scanner at the Brain Imaging Center of Southwest University. Scanning consisted of 5 runs. The first run acquired the high-resolution T1 images. The parameters were as follows: FOV read (256 mm), slices per slab (192), resolution (1 × 1 × 1 mm^3^), TR (2,530 ms), TE (2.98 ms), TI (1,100 ms), and flip angle (7°). The following 4 runs acquired the T2* image. Notably, to acquire the T2* images, we used multi-slice accelerating technology (MSA), which can significantly improve the temporal resolution of the imaging data (Frost, 2015). With the advantages of MSA, we acquired the functional imaging data at 2.5 × 2.5 × 2.5 mm^3^ resolution with a TR only lasting for 1 s. The higher temporal resolution of the functional data helped us obtain a more detailed dynamic processing process of tonal working memory. The parameters used in the T2* scanning were as follows: acceleration factor slice (4), FOV read (195 mm), TR (1,000 ms), TE (30 ms), flip angle (32°), slices (56), and resolution (2.5 × 2.5 × 2.5 mm^3^).

We used FreeSurfer (Fischl et al., 1999b) to implement data preprocessing. First, we used the function “recon-all” (Collins et al., 1994; Dale et al., 1999; Fischl et al., 1999a,b) to reconstruct the cortex with a T1-weighted image. In this step, data processing procedures such as motion correction, Talairach transform, skull strip, white mater segmentation, spherical registration, and cortical parcelation were performed. Second, we used the function “preproc-sess” (Cox and Jesmanowicz, 1999) to preprocess the functional data. In this step, processing procedures such as registration template, motion correction (with motion parameters), anatomical-functional registration, mask creation, intensity normalization (global signal regression), resampling to common space, and spatial smoothing were performed. By preprocessing, the functional data were registered to a high-resolution surface template of fsaverage, which was obtained by the spherical alignment of 40 participants (Wu, 2018). Fsaverage consists of two hemispheres, both of which have 163,842 vertices. It has an inflated form that can significantly benefit visualization. Our following analyses were implemented based on this inflated template.

### Surface-space-based MVPA

In this study, we proposed an sf-MVPA method to reveal the distinction among local cortical activity patterns under different conditions. The surface space of fsaverage has a very fine spatial resolution. We calculated the distances between the vertices and found that the average distance between a vertex and its nearest vertex is 0.542 mm in the left hemisphere and 0.545 mm in the right hemisphere, which is much finer than the spatial resolution of our original functional data. If we directly implemented sf-MVPA in this space, the computational complexity will significantly increase.

**FIGURE 2:**
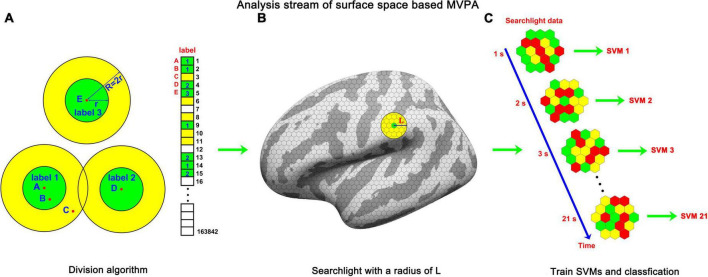
The analysis stream of sf-MVPA. **(A)** Double radius dividing method. We used this method to divide the template of fsaverage into labels as evenly as possible. Dividing was implemented according to the numerical order of the vertices. For a vertex (e.g., points A, D, and E) that was not grouped into a label, the vertices whose distances from the vertex were shorter than r were grouped into a label and painted green. The vertices whose distances from the vertex were longer than r but shorter than R (R = 2r) were painted yellow. For vertices that were painted color (e.g., points B and C) by the painting process of the former vertices, the painting and grouping process was forbidden. The large circle guarantees that the labels will not overlap. After the iteration process, the vertices painted yellow were grouped into the nearest label. **(B)** Searchlight on the surface space. The grid on the surface represents the labels. The yellow circle represents the searchlight. **(C)** The frame-by-frame training and classification process. The hexagons represent labels and the color on them represents the averaged strength of BOLD signal.

To address the problem of computational complexity, we proposed a double radius dividing method to downsample the surface space of fsaverage. The core idea of this method is to divide the surface of fsaverage into small pieces that share a resolution similar to that of the original functional data as evenly as possible. In this article, these pieces were named labels. The surface space of fsaverage is an irregular sphere, which increases the difficulty of uniform subdivision. As illustrated in Figure 2A, the subdivision was implemented in the numerical order of the vertices. The distances between the first vertex (point A) and all remaining vertices were calculated. Vertices whose Euclidean distance from point A was less than a 0.9-mm radius (r) were grouped into label 1. When a vertex was grouped into a label, it was painted green. Meanwhile, vertices whose Euclidean distance from point A was less than 1.8 mm (double radius, R) but larger than 0.9 mm were painted yellow. The double radius R was used to prevent the overlapping of adjacent labels. This painting and grouping operation was iterated for all remaining vertices under the condition that the vertex under processing had not been painted any color by the former processes. For example, vertex 2 (Point B) and vertex 3 (Point C) were painted green and yellow, respectively, by the operation of the first vertex. Therefore, the painting and grouping operation was not applied to these two vertices. Vertex 4 (Point D) and vertex 5 (Point E) were not painted by former processes; thus, the painting and grouping operation was applied to them. After the iteration process was completed, the vertices that had not been grouped into a label (vertices painted yellow) were grouped into the nearest label according to the Euclidean distances between them and the center positions of each label. Then, the label replaced the vertex as the basic unit of the surface space. Using this method, we downsampled the surface space of fsaverage from 163,842 to 11,895 (left hemisphere) and 11,751 (right hemisphere), with approximately 14 vertices per label. The average Euclidean distance between a label and its nearest label (center to center) was 2.08 mm (left hemisphere) and 2.10 mm (right hemisphere), indicating that after downsampling, the surface space was in a comparable resolution with the original functional data (but still smaller than the original resolution). Then, the functional data were downsampled to the label space by averaging the blood oxygen level-dependent (BOLD) signals of vertices inside labels.

The following steps were similar to those of volume space-based MVPA. For each label, a sphere (10 mm in radius, as illustrated in Figure 2B) around the center of the label was constructed. Labels whose center was located inside the sphere constitute the searchlight of the central label. Because the size of the radius (10 mm) of a sphere is much smaller than the size of the template of fsaverage, whose major axis (Y-axis) is 212 mm and minor axis (X-axis) is 80 mm, the searchlight selected by this method is close to a circle on a plane. A searchlight includes 36–91 labels. The fluctuation of the numbers of labels included in a searchlight mainly stems from the non-uniformity of the subdivision and the irregular curvature of the template of fsaverage.

**FIGURE 3:**
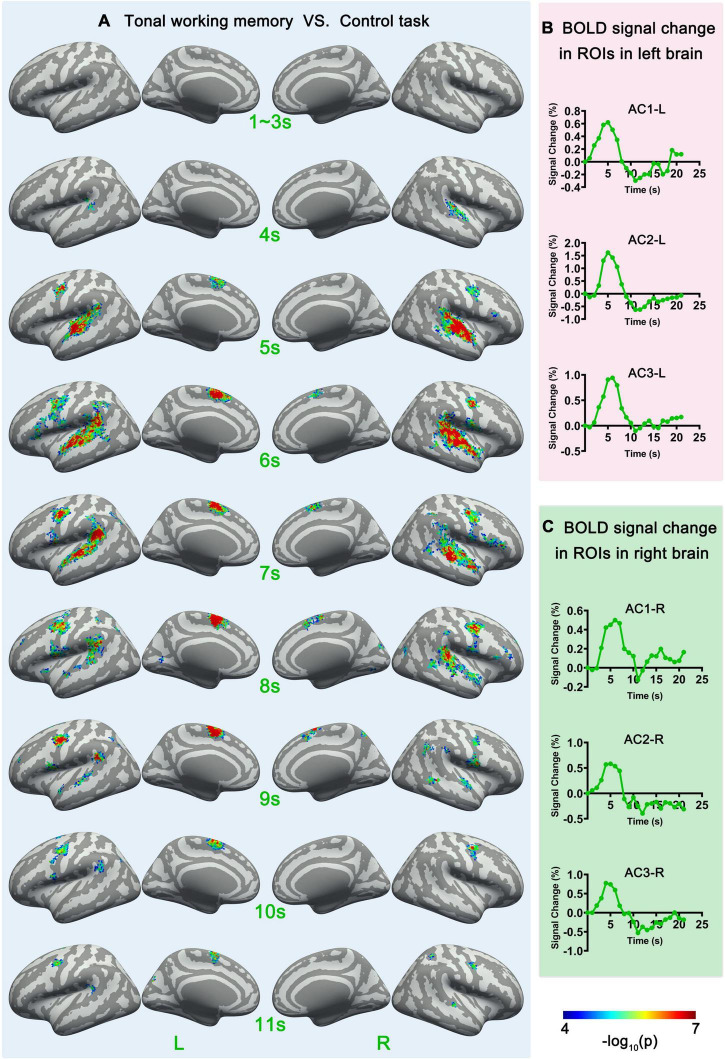
Sf-MVPA analysis results of 1–11 s and BOLD curves of 6 ROIs. **(A)** The sf-MVPA results of 1–11 s of tonal working memory vs. control task. **(B)** Average BOLD curves of 3 ROIs in the left hemisphere. **(C)** Average BOLD curves of 3 ROIs in the right hemisphere. AC, auditory cortex.

**FIGURE 4:**
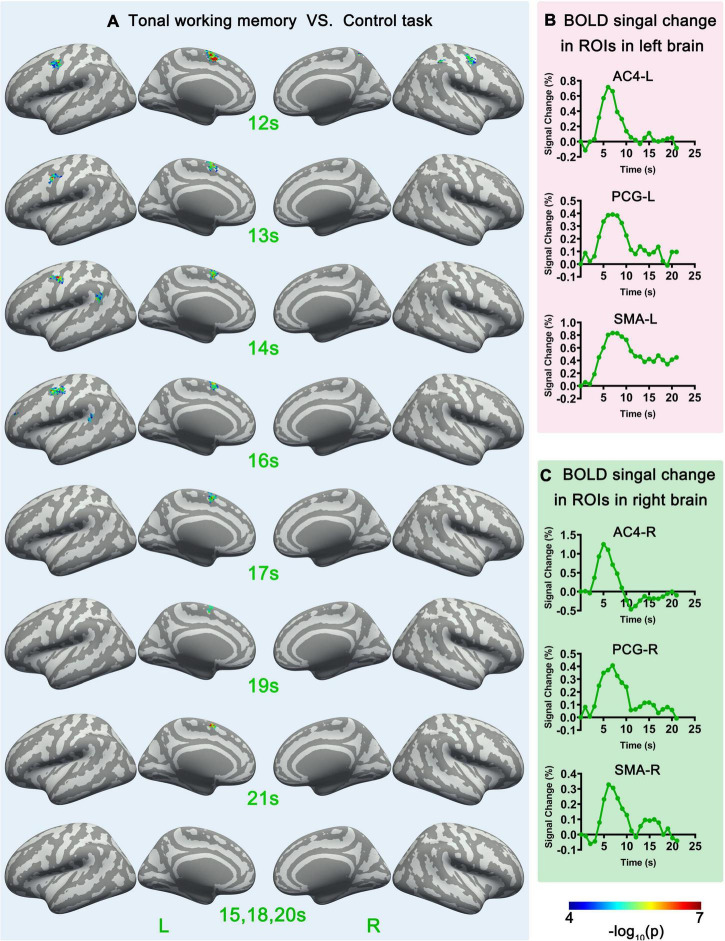
Sf-MVPA analysis results of 12–21 s and BOLD curves of 6 ROIs. **(A)** The sf-MVPA results of 12–21 s of tonal working memory vs. control task. **(B)** Average BOLD curves of 3 ROIs in the left hemisphere. **(C)** Average BOLD curves of 3 ROIs in the right hemisphere. AC, auditory cortex; PCG, precentral gyrus; SMA, supplementary motor area.

Subsequently, we analyzed the local cortical activity pattern distinction between the tonal working memory task and the control task. A threefold cross-validation was applied. The data from two runs served as the training set, and the remaining run served as the testing set. The classification accuracies of the three testing sets were averaged and stored in each label. We used a multiclass error-correcting output code (ECOC) model (Escalera et al., 2008, 2009) to train and classify the data. This model was encapsulated in MATLAB and worked together with support vector machine (SVM) binary learners (Allwein et al., 2000). The number of trials of tonal working memory was four times that of the control condition, which would lead to an imbalance problem (Farquad and Bose, 2012). The imbalance problem refers to when there are many more instances of some classes than others during training, and the classifiers tend to be overwhelmed by the large classes and ignore the small ones. To avoid the imbalance problem, we downsampled the instances of tonal working memory to the same number of trials as the control condition. Under each memory load condition, we randomly selected instances with the number of a quarter of the number of the control condition. After instance downsampling, the functional data were z-score normalized and subjected to the training and classifying procedures, as illustrated in Figure 2C. A second-level random effect analysis (Lee et al., 2011) was implemented by comparing each label’s accuracy to 50% with a unilateral *t*-test. We analyzed the local pattern distinction from the stimulus onset to the end of the delay period, forming a 21-s dynamic process. The results are reported in Figures 3A, 4A with a criterion of p < 0.0001 (significance level of the *t*-test), false discovery rate (Benjamini and Hochberg, 1995) (FDR) *p* < 0.01, and cluster size (label) > 10.

The cluster sizes of the labels meeting the statistical criterion were calculated with an iterative recursive algorithm. First, we set the criterion that if the closest distance (by calculating vertex–vertex distances) between the two labels was less than 1.1 mm, the two labels were judged as adjacent to each other. For every label that met the statistical criterion, the following iterative process was invoked. If a label was not grouped into a cluster, this label and its adjacent labels that met the statistical criterion were grouped into a cluster. Then, the process was recursively invoked by its adjacent labels that met the statistical criterion. This algorithm stopped automatically when all labels that met the statistical criterion were grouped into a cluster. Through this iterative recursive algorithm, cluster sizes were successfully counted.

After the analysis of tonal working memory vs. the control task, we analyzed the local pattern distinction under different tonal working memory loads with the sf-MVPA method. Three changes were applied. First, because the numbers of trials of different memory loads were comparable, downsampling of instances was not applied. Second, in the second-level random-effect analysis, the accuracies of the classification were compared to 25%, corresponding to 4 conditions. Third, instances of the control condition were not included in this analysis.

### Response strength analysis of fMRI data

We used general linear mode (GLM) to explore whether there are brain areas whose activity strength increases as tonal working memory load increases. Using FreeSurfer, the onset times of different conditions (with a duration of 21 s) were convolved with a standard hemodynamic response functional (HRF) curve to construct simulated response curves. These curves entered the GLM, and the regression coefficients were calculated. Then, the second-level random effects were analyzed, and the contrasts between adjacent conditions were compared. The statistical criteria of these comparisons were set to *p* < 0.01 (voxel-wise threshold) and *p* < 0.05 (cluster-wise threshold). GLM analysis was implemented in the standard vertex space of fsaverage.

We also used a linear regression (Chatterjee and Hadi, 1986) to analyze the relation between memory load and neural activity strength. Linear regression analysis was implemented in label space with MATLAB. We constructed the average BOLD response curves of each label under each memory condition by subtracting the average response curve under the control condition from each condition’s average response curve. The last second before the stimulus was set as the baseline (zero point); thus, the curve lasted for 21 s. The average curves across subjects and conditions of 12 return on investments (ROIs) (introduced in the next part) are displayed in Figures 3B,C, 4B,C. The average amplitudes of the response curves under each condition were stored in each label and entered the group-level linear regression analysis between memory load and neural activity strength. The statistical criteria were set to *p* < 0.05 (significance level) and cluster size (label) > 10.

### Functional connectivity analysis

Surface-space-based multivariate pattern analysis of tonal working memory vs. the control condition revealed the dynamic cortical processing process of tonal working memory. During the process, some brain areas, such as the bilateral AC, IPL, PCG, and SMA dynamically participated in the processing of tonal working memory. However, in contrast to the IPL, PCG, and SMA, which have relatively stable peak positions, the peak position of AC varies with time (as shown in Figure 3). Therefore, we choose the three peak positions of AC in the fifth, sixth, and seventh seconds as ROI. Thus, there were 12 ROIs in total. The names and coordinates of these ROIs and the time points from which they were selected are reported in Table 1.

**TABLE 1:** Names and locations of 12 ROIs.

				Talairach coordinates		
Hemisphere	Name	Time point	Region	x	y	Z
Left	AC1	5s	Superior temporal gyrus left	–37.0	–26.1	9.6
	AC2	5s	Superior temporal gyrus left	–49.0	–22.0	6.0
	AC3	7s	Superior temporal gyrus left	–59.5	–16.3	2.1
	AC4	7s	Superior temporal gyrus left	–59.0	–44.2	19.5
	PCG	9s	Precentral gyrus left	–44.5	1.6	36.7
	SMA	8s	Supplementary motor area left	–6.6	12.4	56.1
Right	AC1	7s	Superior temporal gyrus right	53.6	–25.2	0.3
	AC2	6s	Superior temporal gyrus right	43.0	–17.7	–6.1
	AC3	5s	Superior temporal gyrus right	38.1	–25.5	10.7
	AC4	8s	Superior temporal gyrus right	58.5	–28.3	9.3
	PCG	8s	Precentral gyrus right	49.6	–3.1	44.3
	SMA	7s	Supplementary motor area right	7.0	15.1	57.0

We calculated the functional connectivity strength between these ROIs and each label under different memory loads. In each trial, Person correlations of the 21-s BOLD curves between the 12 ROIs and all labels were calculated. It is worth noting that the BOLD curves of the 12 ROIs were average curves of each ROI and their adjacent labels. The correlation coefficients under each memory condition were averaged and stored in each label. A group-level linear regression between the correlation coefficients and memory load was performed for each label and ROI. Brain areas whose functional connectivity strength with an ROI was linearly correlated with the memory load are reported with a criterion of *p* < 0.05 (significance level) and cluster size (label) > 10.

## Results

### Behavioral results

The average response accuracy under the four memory conditions was 75.7% and SD 7.5%. The accuracies under each condition were as follows: Condition 1 (77.6% and SD 12.5%), Condition 2 (84.1% and SD 14.7%), Condition 3 (75.4% and SD 12.4%), and Condition 4 (65.7% and SD 8.7%). The behavioral results are displayed in Figure 1B.

### Sf-MVPA of tonal working memory vs. control task

**FIGURE 5:**
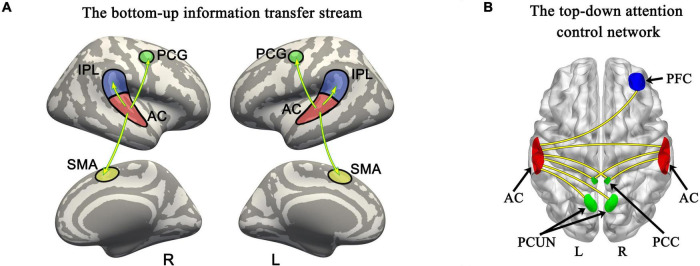
Bottom-up information transfer process and top-down attention control network during tonal working memory. **(A)** The information transfer process during tonal working memory. **(B)** The attention control network during tonal working memory. PCG, precentral gyrus; AC, auditory cortex; IPL, inferior parietal lobule; SMA, supplementary motor area; PFC, prefrontal cortex; PCC, posterior cingulate cortex; PCUN, precuneus.

Surface-space-based multivariate pattern analysis revealed a dynamic cortical processing process of tonal working memory. As shown in Figures 3A, 4A, the cortical processing of tonal working memory started from the 4th second with small area activation in the bilateral AC. Then, during 5–7 s, the bilateral AC was strongly activated and the activation was transmitted to the bilateral IPL, PCG, and SMA. During 8–12 s, activation in the bilateral AC gradually faded, and activations were mainly maintained in the bilateral PCG and SMA. After the 12th second, the activation in the right hemisphere disappeared but the left PCG and SMA remained activated. After the 16th second, the activation of the left PCG disappeared and the left SMA was the only remaining activated brain area. Notably, after the 12th second, the strength and area of the activation of the left hemisphere gradually declined, and at 15, 18, and 20 s, there was no activation of either hemisphere. We summarized the activation sequence and made a summary in Figure 5A.

Notably, neural activity and BOLD signals have a complicated non-linear relationship (Baumann, 2010). Thus, there is a delay and non-linear mapping relationship between the actual occurrence time of neural activity and the time that sf-MVPA can detect changes in local activity patterns. To simplify the results, we directly reported and discussed the sf-MVPA results without considering the delay of HRF.

### Sf-MVPA and response strength analysis of tonal working memory load

**FIGURE 6:**
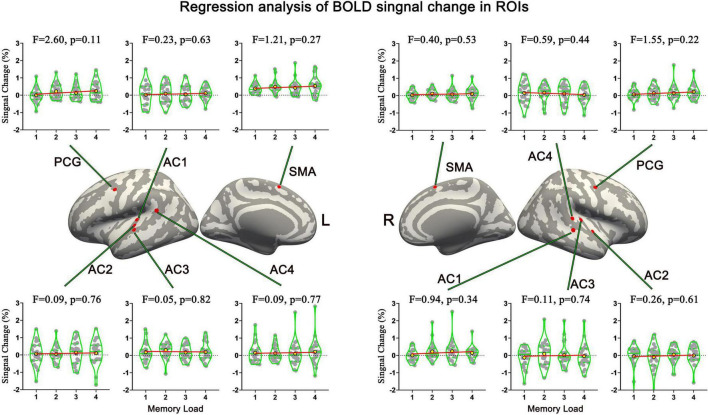
Names and locations and regression analysis results of neural response strength of 12 ROIs. No brain area’s response strength was linearly correlated with memory load. AC, auditory cortex; PCG, precentral gyrus; SMA, supplementary motor area.

Sf-MVPA of tonal working memory load showed that no brain area’s classification accuracy was significantly higher than 25% during the analyzed 21 s, indicating that the local activity patterns under the four memory load conditions did not significantly differ. In addition, GLM analysis showed that no brain area’s response strength significantly differs between adjacent conditions. Furthermore, regression analysis validated the GLM results by showing that no brain area’s response strength was linearly correlated with the memory load. The regression analysis results of the 12 ROIs are displayed in Figure 6.

### Functional connectivity analysis

**FIGURE 7:**
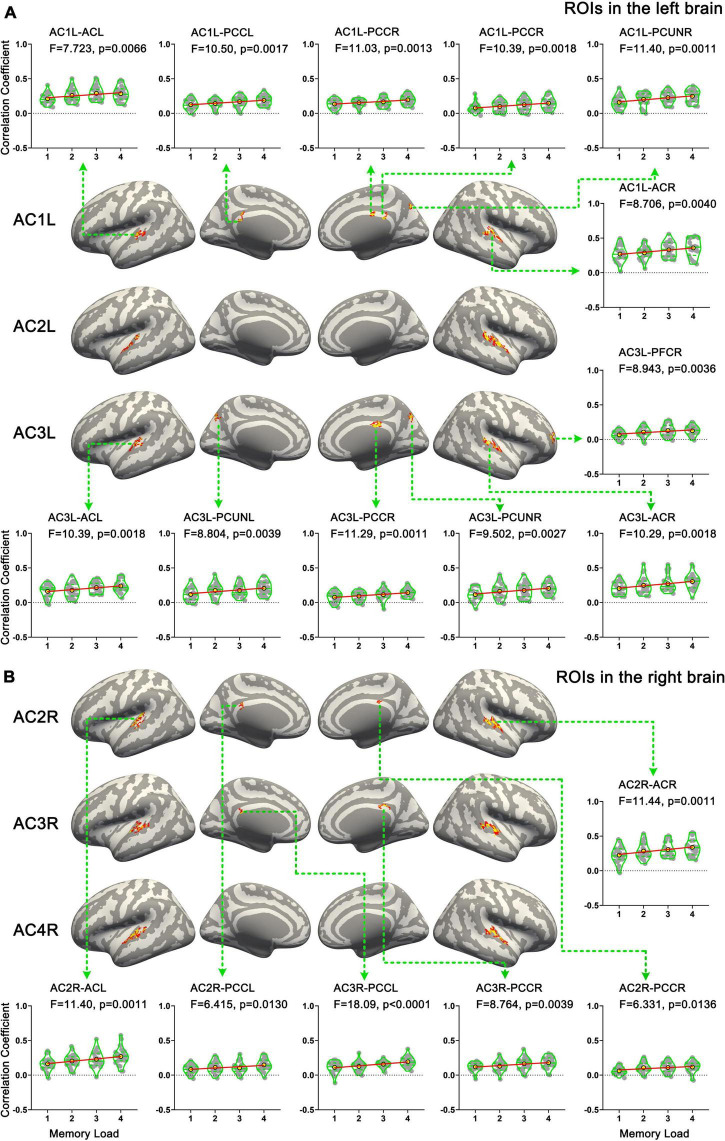
Results of functional connectivity analysis. **(A)** Brain areas whose functional connectivity strength with ROIs in the left hemisphere was linearly correlated with memory load. **(B)** Brain areas whose functional connectivity strength with ROIs in the right hemisphere was linearly correlated with memory load. AC, auditory cortex; PFC, prefrontal cortex; PCUN, precuneus; PCC, posterior cingulate cortex.

In total, six ROIs (three in each AC) manifested an increased functional connectivity with cortical areas as the memory load increased. In the left AC, as the memory load increased, AC1L showed an increased connectivity with bilateral AC, bilateral PCC, and the right precuneus; AC2L showed an increased connectivity with bilateral AC; and AC3L showed an increased connectivity with bilateral AC, the right PCC, the bilateral precuneus, and the right PFC. In the right AC, AC2R showed an increased connectivity with bilateral AC and bilateral PCC; AC3R showed an increased connectivity with bilateral AC and bilateral PCC; and AC4R showed an increased connectivity with bilateral AC. The results of functional connectivity analysis along with the results of regression analysis of the peak labels are displayed in Figures 5B, 7A,B and Table 2.

**TABLE 2:** Results of functional connectivity analysis.

				Talairach coordinates		
ROI	-log_10_ (p)	Size (mm^2^)	Region	x	y	Z
AC1L	2.178	278.0	Superior temporal gyrus left	–51.4	–20.2	2.6
	2.775	57.3	Posterior cingulate cortex left	–6.1	–39.0	22.6
	2.393	259.6	Superior temporal gyrus right	46.2	–23.9	7.1
	2.674	117.0	Superior temporal gyrus right	63.9	–29.5	4.0
	2.754	35.5	Posterior cingulate cortex right	2.2	–31.4	24.2
	2.887	34.6	Posterior cingulate cortex right	6.9	–15.1	28.1
	2.964	93.4	Precuneus right	10.4	–66.1	39.8
AC2L	2.120	156.4	Superior temporal gyrus left	–50.6	–12.4	1.0
	2.705	92.0	Superior temporal gyrus left	–44.8	–25.9	6.3
	3.981	740.8	Superior temporal gyrus right	65.7	–25.0	4.9
AC3L	2.415	143.7	Superior temporal gyrus left	–10.7	–70.1	43.6
	2.753	297.0	Superior temporal gyrus left	–38.5	–33.1	8.8
	2.445	158.5	Prefrontal cortex right	37.2	49.5	8.8
	2.533	88.9	Prefrontal cortex right	25.0	48.7	11.0
	2.565	168.3	Precuneus right	12.2	–66.2	40.9
	2.733	261.6	Superior temporal gyrus right	51.9	–12.1	2.8
	2.904	133.1	Superior temporal gyrus right	62.9	–25.2	6.8
	2.941	91.6	Posterior cingulate cortex right	7.7	–24.9	27.3
AC2R	1.885	37.9	Posterior cingulate cortex left	–8.2	–34.4	27.7
	2.233	119.4	Superior temporal gyrus left	–62.6	–17.2	0.0
	2.965	309.8	Superior temporal gyrus left	–43.3	–23.9	9.3
	1.865	27.5	Posterior cingulate cortex right	7.6	–25.2	29.6
	2.972	341.6	Superior temporal gyrus right	48.2	–20.0	5.9
	3.038	165.8	Superior temporal gyrus right	65.9	–23.4	3.3
AC3R	2.552	480.5	Superior temporal gyrus left	–59.6	–26.3	6.7
	4.288	32.3	Posterior cingulate cortex left	–4.1	–37.0	22.9
	2.406	64.5	Posterior cingulate cortex right	5.7	–31.5	28.4
	2.880	496.4	Superior temporal gyrus right	65.9	–23.4	3.3
AC4R	2.690	88.1	Superior temporal gyrus left	–54.9	–31.1	8.4
	2.780	505.0	Superior temporal gyrus left	–47.1	–18.2	5.6
	3.552	538.7	Superior temporal gyrus right	49.7	–22.3	5.7

Brain areas whose functional connectivity strength with ROIs was linearly correlated with memory load.

## Discussion

In this article, we studied the dynamic processing process of tonal working memory and the cortical correlates of tonal working memory load. Our results showed that (i) the cortical processing process of tonal working memory is a bottom-up information transfer process; (ii) in all analyzed 21 s, no brain area’s local activity pattern was distinguishable among different memory loads; (iii) no brain area’s local activity strength significantly increased as memory load increased; and (iv) the functional connectivity strength between the AC and an attention control network, consisting of the PFC, PCC, and precuneus was linearly correlated with tonal working memory load.

### The original sf-MVPA approach

The existing sf-MVPA approaches (Oosterhof et al., 2010; Chen, 2011) have the problem of repetitive calculation. In this article, we proposed a double radius downsampling algorithm to solve this problem. Using this algorithm, we successfully downsampled the surface space of fsaverage from the vertex space, which contains 163,842 vertices in each hemisphere, to the label space, which contains 11,895 labels in the left hemisphere and 11,751 labels in the right hemisphere. Because the computational complexity of sf-MVPA linearly correlates with the number of units in a space, our method can significantly reduce the computational complexity by an order of magnitude. Moreover, because the resolution of the label space is still less than the resolution of the original functional data, our method does not compromise the resolution. Furthermore, our approach directly extracts local activity pattern data from the label space, which is more intuitive and can retain the proportion of local activity patterns.

In summary, compared to the existing sf-MVPA approaches (Chen, 2011; Oosterhof et al., 2010), our sf-MVPA approach significantly decreases the computational complexity, does not compromise the resolution, and applies a more intuitive and reasonable searchlight data extracting method.

### Dynamic cortical processing process of tonal working memory

Compared to GLM, a significant advantage of MVPA is that it can analyze fMRI data frame by frame. Based on this advantage, our sf-MVPA method successfully revealed a dynamic cortical processing process of tonal working memory. During this process, tonal information is sequentially transmitted to the AC, IPL, PCG, and SMA, all of which are important parts of the neural basis of tonal working memory (Chatterjee and Hadi, 1986; Benjamini and Hochberg, 1995; Baumann, 2010; Oosterhof et al., 2010; Lee et al., 2011; Farquad and Bose, 2012), and then sequentially disappears in these areas, forming a bottom-up information transfer process. The order and duration of the neural activity indicated the role of the brain areas in maintaining tonal working memory. Tonal information was first transmitted to the AC, but the information was not maintained in the AC for a long time and was then transmitted to higher level cortices (i.e., the IPL, PCG, and SMA) just 1 s later. Thus, the role of the AC in this process is more like a perception and transmitting station. This presumption is consistent with animal experiments (Bigelow et al., 2014; Yu, 2021) that auditory working memory information is only maintained in the AC in the early stage of the retention period. After the 5th second, tonal information is transmitted to and sustainably maintained in the IPL, PCG, and SMA. The sustained activity of a given brain area in the maintenance period is generally considered the neural basis of working memory (Kumar, 2016). Thus, the IPL, PCG, and SMA played a higher role in maintaining tonal information in this network. Furthermore, a more detailed hierarchical structure seems to exist in these three areas. With the tonal information represented in the local patterns of IPL and PCG disappearing after the 16th second, the left SMA was the only brain area that carried tonal working memory in the late stage of the maintenance period. Thus, the left SMA was the only brain area that carried tonal working memory throughout the whole maintenance period, indicating its core role in tonal working memory. The SMA has been found to play an important role in maintaining auditory working memory. In a verbal working memory study (Huang et al., 2013), activity in the bilateral SMA was found to increase with memory load. In an fMRI study (Hoddinott et al., 2021) comparing the neural basis of verbal and rhythmic working memory, the left SMA was found to be activated during the maintenance of verbal working memory. When studying the neural basis of content-specific (pitches) working memory (Uluç et al., 2018), the bilateral SMA was found to carry information on the working memory of pitches.

In addition to the left SMA, the left PCG and IPL were also left-side laterally activated during the middle stage of the maintenance period, showing the left laterality of the network. Taken together, the dynamic bottom-up information transfer stream in the cortex supported the maintenance of tonal working memory.

Utilizing the good spatial and temporal resolution of our sf-MVPA method, we successfully demonstrated the dynamic processing process of tonal working memory. To the best of our knowledge, this study is the first to clearly demonstrate this process, showing the promising prospects of our sf-MVPA method.

### Neural mechanisms underlying tonal working memory load

Tonal working memory has been studied from the perspective of local activity intensity and local activity pattern (Gaab et al., 2003; Nolden, 2013; Kumar, 2016; Uluç et al., 2018). However, the neural basis of tonal working memory load showed a different perspective. Our sf-MVPA results showed that no brain area’s local cortical activity pattern was distinguishable among different memory loads. In addition, GLM and linear regression analysis showed that no brain area’s response strength increased as the memory load increased. Finally, our functional connectivity analysis showed that functional connectivity from the left AC to the right AC, bilateral PCC, bilateral precuneus, and right PFC and the connectivity from the right AC to the left AC and bilateral PCC were linearly correlated with tonal working memory load.

According to Baddeley’s working memory model (Baddeley, 1986, 2000), a working memory system includes a master component (the central execute) and some slave components, such as the phonological loop, visuospatial sketchpad, and episodic buffer. The central execute coordinates and integrates the demands of the slave components by allocating limited attention resources to each component according to the current goal (Pechmann and Mohr, 1992; D’Esposito and Postle, 2015; Baddeley, 2020). In addition to Baddeley’s model, recently, popular state-based models [refer to ref. (D’Esposito and Postle, 2015) for a review] proposed a similar opinion. These models posit that working memory relies on a top-down attention selection mechanism and that attention selection of mental representations brings them into working memory (Cowan, 1998; D’Esposito and Postle, 2015). Despite the differences, both Baddeley’s and state-based models stressed the interaction between top-down attention control and memory storage units during working memory. It has been widely accepted that the PFC plays an important role in the top-down attention control mechanism during working memory (Funahashi, 2017; Nee and D’Esposito, 2018). In addition to the PFC, the PCC and precenus were found to be associated with attention control. In an fMRI study exploring the domain-general network of working memory, Li et al. (2014) found that the PCC and precuneus played a role in allocating attention resources to memory contents. In another fMRI study investigating spatial working memory, Bledowski et al. (2009) found that the PCC and precuneus are related to the process of allocating attention to specific items. In addition, as important hubs of the default mode network, the PCC and precuneus are believed to be essential for the executive control of attention (Andrews-Hanna et al., 2014; Leech and Sharp, 2014). Taken together, it can be concluded that the PFC, PCC, and precuneus constitute at least part of the top-down attention control network in working memory.

Our results showed that the interactions between the AC and this top-down attention control network (i.e., the right PFC, PCC, and precuneus) were linearly correlated with tonal working memory load. Considering this result and working memory models (Baddeley, 1986, 2000; D’Esposito and Postle, 2015) together, it can be inferred that as the memory load of the current task increases, by strengthening the functional connectivity between the attention control network and task-related cortices, more attention is focused on the current task-related working memory, allocating more limited working memory resource to the current task and, thus, increasing the ability to retain more items in working memory. Thus, the neural mechanisms associated with tonal working memory load do not arise from changes in local cortical activity patterns or changes in the local cortical activity strength, but from top-down attention control of the working memory system. Although similar research on tonal studies is lacking, this conclusion is consistent with related studies in the visual domain. In an LFP study (Pinotsis et al., 2019) involving monkeys, visual memory load was found to be correlated with the connectivity between the PFC and the visual sensory cortex. In an EEG and MEG study (Palva et al., 2010), synchrony among the frontoparietal regions, which are known to underlie executive and attentional functions, was found to be correlated with memory load. Furthermore, the interactions among the PCC, precuneus, and angular area were found to be correlated with visual working memory load in an fMRI study (Vatansever et al., 2017). All these studies revealed the connection between memory load and the functional connectivity between the attention control network and task-related cortices.

Taken together, our results show that the realization of tonal working memory involves the following two information streams: the bottom-up information transfer stream, which perceives and transfers tonal information to memory storage units, and the top-down attention control stream, which allocates working memory resources by adjusting the connectivity strength between the AC and the attention control network. The cooperation of these two streams constitutes the neural basis underlying tonal working memory.

In addition, as stated earlier, the dynamic processing process of tonal working memory showed left-sided laterality. The results of functional connectivity analysis also showed that the left AC played a more central role than the right AC. Taking both of these results into account, it can be concluded that the tonal working memory system shows left laterality. This finding may contradict some former studies (Lee et al., 2011; Albouy, 2019; Sihvonen, 2019), but is also supported by some other studies (Gaab et al., 2003; Hyde et al., 2011; Schaal, 2015).

## Conclusion

In this article, we studied the neural basis underlying tonal working memory load. We found that the realization of tonal working memory requires the support of two information streams, one bottom-up information transfer stream and one top-down attention control stream. Meanwhile, the strength of the top-down attention control stream was modulated by memory load, which supported the working memory model of Baddeley (Baddeley, 1986, 2000). Our study revealed the complexity of the neural basis of tonal working memory.

## Data availability statement

The data and code used in this manuscript and the necessary information to analyze the data are available at https://pan.baidu.com/s/1yzuHuYeLRlLJllPuDuyAVA, extracting code: liqi.

## Ethics statement

The studies involving human participants were reviewed and approved by the Ethics Committee of Southwest University. The patients/participants provided their written informed consent to participate in this study.

## Footnotes

1
https://ww2.mathworks.cn/

2
http://lilypond.org/

3
https://www.adobe.com/cn/products/audition.html

4
https://visualstudio.microsoft.com

## Abbreviations

STG: superior temporal gyrus
SMG: supramarginal gyrus
IFG: inferior frontal gyrus
PCG: Precentral gyrus
SMA: Supplementary motor area
PMC: Premotor cortex
IPL: Inferior parietal lobe
PT: Planum temporale
AC: Auditory cortex
EEG: Electroencephalography
MEG: Magnetoencephalography
MVPA: Multivariate pattern analysis
fMRI: Functional magnetic resonance imaging
LFP: Local field potential
PFC: Prefrontal cortex
PCC: Posterior cingulate cortex
ECOC: Multiclass error-correcting output codes
SVM: Support vector machine
FDR: False discovery rate
GLM: General linear mode
BOLD: Blood oxygen level dependent.
